# Effects of thymol and eugenol supplementation on reproductive performance, egg quality, and offspring health in broiler breeders

**DOI:** 10.1016/j.psj.2026.107193

**Published:** 2026-05-27

**Authors:** Haojian Sun, Peng Sun, Xinran Zhang, Okasha Hamada, Linglian Kong, Zhigang Song

**Affiliations:** a Key Laboratory of Efficient Utilization of Non-Grain Feed Resources, College of Animal Science and Technology, Shandong Agricultural University, Taian, Shandong 271018, China; b Animal Production Department, Faculty of Agriculture, Benha University, Moshtohor 13736, Egypt; c Office of Assessment, Jining Polytechnic, Jining, Shandong 272037, China

**Keywords:** Essential oil, Broiler breeder, Maternal effect, Intestinal health, Oxidative stress

## Abstract

This study investigated the effects of a thymol and eugenol blend on broiler breeders and maternal effects on offspring health. A total of 60,000 55-wk-old Hubbard broiler breeders were allocated to 8 houses (n = 4 houses/group, 7,500 birds/house) and assigned to either a basal diet or a treatment supplemented with 12 mg/kg thymol and 4.5 mg/kg eugenol for 60 d. Breeder samples were collected at the end of the trial, and fertile eggs were incubated. A total of 144 one-day-old chicks (72 per group) were selected for a 7-day rearing trial. Results indicated that supplementation increased the percentage of settable eggs, eggshell strength, and Haugh units (*P* < 0.05). In breeders, the essential oil blend improved breeder intestinal morphology, characterized by increased villus height (*P* = 0.002) and the villus height-to-crypt depth ratio (*P* = 0.012), while concurrently enhancing systemic antioxidant capacity and reducing pro-inflammatory cytokines in the ovary and uterus (*P* < 0.05). In offspring, the average daily feed intake of 7-day-old chicks was significantly reduced (*P* = 0.036); however, average daily gain remained unaffected (*P* > 0.05), indicating improved feed utilization. Furthermore, serum and tissue analyses revealed sustained maternal benefits: progeny at 1 and 7 d of age showed reduced pro-inflammatory cytokines and increased antioxidant enzyme activities in the serum, liver, and heart (*P* < 0.05). These benefits demonstrated a maternal transmission effect, enhancing the early-life antioxidant and anti-inflammatory status of offspring. These findings suggest that this essential oil blend is a viable nutritional strategy for optimizing productivity throughout the breeder-progeny cycle.

## Introduction

The global poultry industry is currently confronting a critical and increasingly severe challenge: reducing reliance on antibiotic growth promoters while maintaining optimal production performance. This trend is primarily driven by the growing prominence of drug resistance issues and increasing consumer demand for natural farming practices (Haque et al., 2020; de Mesquita Souza Saraiva et al., 2022; Rahman et al., 2022). In this context, plant-derived feed additives have emerged as a suitable alternative. Among these, essential oil components, represented by thymol and eugenol, have demonstrated significant potential in enhancing poultry production performance and addressing reproductive disorders and metabolic stress encountered in broiler breeder production (Oni et al., 2024), owing to their well-documented antimicrobial, antioxidant, and anti-inflammatory properties (Mnisi et al., 2023).

As the cornerstone of the poultry industry, the nutritional and health status of broiler breeders directly influences egg production, fertilization rate, hatchability, and the quality of the offspring chicks (Yaripour et al., 2018; Noetzold and Zuidhof, 2025). The unique physiological demands of broiler breeders, combined with necessary feed restriction measures to prevent obesity, collectively induce metabolic challenges that may compromise reproductive performance (Decuypere et al., 2010; Korver, 2023). Furthermore, the high metabolic load associated with high egg production can induce oxidative stress, impairing ovarian function and eggshell quality, while persistent chronic inflammation may weaken nutrient absorption and immune competence. These factors collectively contribute to reduced hatchability and chick quality, highlighting the need for nutritional strategies to enhance the antioxidant capacity and modulate inflammatory responses in breeder hens (Oke et al., 2024). Thymol and eugenol exhibit multiple mechanisms of action in this regard: both demonstrate significant antimicrobial activity against common poultry pathogens, while also improving intestinal structure and promoting nutrient absorption (Silva Júnior et al., 2020; Aljuwayd et al., 2023). Their antioxidant properties help mitigate the negative effects of oxidative damage on the reproductive system (Basiouni et al., 2023). Therefore, the combined use of thymol and eugenol may enhance the overall reproductive performance of broiler breeders through the synergistic regulation of three major stressors—microbial, oxidative, and inflammatory.

Maternal nutrition is a critical factor influencing the early development of offspring in poultry, with breeder egg composition serving as a key medium for transmitting nutritional effects (Dixon et al., 2016). Essential oils derived from plants contain antioxidant and immunomodulatory components that may be transferred from the hen to the egg, thereby potentially supporting embryonic development and the early physiological status of chicks. Elevated levels of antioxidant substances in the yolk are particularly beneficial for improving the growth and development of neonatal chicks during the critical first week post-hatch, a period during which they remain highly dependent on residual yolk nutrients to sustain growth and immune defense (Cherian, 2015). Concurrently, the proper development of the intestinal architecture during this early stage establishes the foundation for lifelong production performance, making this phase a crucial window for modulating overall flock health and productivity (Jha et al., 2019). Therefore, the potential of supplementing maternal diets with thymol and eugenol to actively regulate these developmental processes warrants further investigation.

Although research on the application of plant-derived feed additives in poultry nutrition is increasing, existing findings have primarily focused on the effects of single active compounds (such as thymol, eugenol, carvacrol, and artemisinin) in broilers or laying hens (Rossi et al. 2020; Gholami‐Ahangaran et al. 2022). However, combinations of these phenolic compounds often exhibit superior bioactivity through multi-target modes of action, yet systematic investigations into their combined effects—especially regarding the 'broiler breeder-offspring' axis throughout a complete production cycle—remain limited. Given the potential for complementary actions in stabilizing the liver-gut-immune axis, evaluating an optimized blend of thymol and eugenol provides greater practical relevance for commercial production than evaluating isolated components. Therefore, this study evaluated the effects of a commercially-validated dietary supplementation of thymol (12 mg/kg) and eugenol (4.5 mg/kg) on reproductive performance (laying rate, fertility rate, and hatchability), egg quality traits, and systemic antioxidant and inflammatory status in broiler breeders. Furthermore, the transgenerational benefits on growth performance, antioxidant capacity, and inflammatory status of offspring at 1 and 7 days of age were assessed. The objective of this study was to evaluate the potential of this essential oil blend to enhance the reproductive performance of broiler breeders and improve chick quality through transgenerational transmission.

## Materials and methods

### Essential oil

The thymol (purity ≥ 98%) and eugenol (purity ≥ 80%, derived from clove leaf oil,) used in this study were supplied by PlusVet Animal Health Co., Ltd. (Qingdao, China).

### Study design

A total of 60,000 55-wk-old Hubbard Lean-Five broiler breeder chickens (male-to-female ratio of 1:10) were utilized in this 60-d study. The feeding trial was initiated with hens at 55 wk of age, representing the late-lay period. This stage was specifically chosen to circumvent the 'ceiling effect' that often occurs during peak production when birds are in optimal physiological condition, which could otherwise obscure the potential benefits of the essential oil blend. The birds were bred and raised by Shandong Hekangyuan Group Co., Ltd. (Shandong, China). The breeder feeding trial, egg incubation, and offspring rearing trial were conducted at the No. 1 Lanling Breeder Farm (Lanling, Shandong, China) belonging to the same company. The breeder chickens were randomly allocated into two experimental groups, each consisting of four replicates (individual houses), with 7,500 birds per replicate serving as the experimental unit. The two groups were fed either a basal diet (**CON**) or a basal diet supplemented with 12 mg/kg thymol and 4.5 mg/kg eugenol (**EO**). The specific inclusion levels and ratio of this essential oil blend were determined as an optimized commercial formulation, based on five years of longitudinal production experience and empirical field data from PlusVet Animal Health Co., Ltd. (Qingdao, China), which identified this dosage as optimal for maintaining flock health and reproductive consistency. Furthermore, previous studies (Özek et al., 2011; Giannenas et al., 2014; Gholami-Ahangaran et al., 2022) have reported synergistic bioactivities of thymol and eugenol within similar concentration ranges. By utilizing this validated dosage, the current study aimed to provide a high-fidelity assessment of the blend’s transgenerational efficacy under standardized, large-scale production conditions.

Throughout the trial, chickens were housed in automated environmental control chambers maintained at 20 ± 3°C and 65–75% relative humidity under a 16-hour light/8-hour dark photoperiod, with ad libitum access to feed and water. The basal diet (Supplementary Table S1) was formulated to meet the nutrient requirements of Hubbard Lean-Five broiler breeders as recommended in the Lean-Five Breeder Management Guide (Hubbard, 2021), and was also consistent with the nutritional specifications stipulated in the Feeding Standard of Chicken NY/T 33-2004 (Ministry of Agriculture of the People's Republic of China, 2004). Metabolizable energy (**ME**) and available phosphorus were calculated based on the Table of Feed Composition and Nutritive Values (China Feed Database, 2020). Feed samples were collected and analyzed for amino acid profiles using a fully automated analyzer (L-8900, Hitachi Ltd., Tokyo, Japan), strictly following the Determination of Amino Acids in Feeds (GB/T 18246-2019, China National Standard, 2019). Calcium content was determined by titration assay, in compliance with the China National Standard (GB/T 6436-2018, China National Standard, 2018). The animal study protocol was reviewed and approved by the Institutional Animal Care and Use Committee of the Faculty of Animal Science and Technology, Shandong Agricultural University (Approval No. SDAUA-2023-35).

### Sample collection

On day 60 of the trial, three roosters and three hens were randomly selected from each replicate for sample collection. Blood was drawn from the wing vein sinus using a sterile syringe and placed into a 5 mL centrifuge tube without anticoagulant for 1 hour. The samples were then centrifuged at 3,000 × *g* at 4 °C for 15 min. Serum was separated and stored at −20 °C for subsequent analysis. After blood collection, the hens were euthanized by cervical dislocation and immediately dissected. A 2 cm segment from the mid-jejunum was excised, gently rinsed with PBS to remove luminal contents, and fixed in 4% paraformaldehyde for morphological analysis. Tissue samples from the liver, ovary, uterus, and jejunum were collected and stored at −80 °C for molecular and biochemical assays.

### Reproductive performance

The measurement and calculation of reproductive performance were conducted in accordance with the Agricultural Industry Standard of the People’s Republic of China, "Performance Terminology and Measurements for Poultry " (NY/T823-2020). The laying rate was calculated from the total number of eggs laid, and the average egg weight was determined from the total egg mass. The feed conversion ratio for eggs (**FCRe**) was calculated as feed consumption divided by the total number of eggs produced. For breeding hens, the number of settable eggs was recorded, and the percentage of settable eggs was calculated. The fertility was determined as the number of fertilized eggs divided by the number of eggs set. The hatchability of fertile eggs (**HF**) was calculated as the number of hatched chicks divided by the number of fertilized eggs, while the hatchability of settable eggs (**HS**) was calculated as the number of hatched chicks divided by the number of eggs set. The percentage of healthy day-old chicks (**HCP**) was determined as the number of healthy chicks divided by the total number of hatched chicks.

### Egg quality

On day 60 of the trial, 10 eggs were randomly selected from each replicate group for egg quality assessment. The eggs were placed horizontally, and their long axis and short axis were measured using a digital vernier caliper. The egg shape index was calculated according to the formula: short axis/long axis. Eggshell lightness (L*), red-green chromaticity (a*), and yellow-blue chromaticity (b*) were measured using a colorimeter (CR-10 Plus, Konica Minolta Inc, Japan). Eggshell thickness was measured using a thickness gauge (ETG-1061, Robotmation Corporation, Japan), and eggshell strength was assessed using an eggshell strength tester (EFG-0.5.3, Robotmation Corporation). Albumen height, yolk color, and Haugh unit (HU) were measured using a multifunctional egg quality analyzer (Orka Food Technology, Israel).

### Biochemical indices in serum, liver, ovary, uterus, and jejunum tissues

The total antioxidant capacity (**T-AOC**), reduced glutathione (**GSH**) content, and the activities of catalase (**CAT**), superoxide dismutase (**SOD**), and glutathione peroxidase (**GSH-Px**) in various tissues were measured using kits provided by the Nanjing Jiancheng Bioengineering Institute (Nanjing, China). Levels of tumor necrosis factor-alpha (**TNF-α**), interleukin-1β (**IL-1β**), and interleukin-6 (**IL-6**) were determined using enzyme-linked immunosorbent assay kits (MLBIO Co., Shanghai, China). All procedures were strictly performed according to the manufacturer’s instructions, with intra-assay and inter-assay CV not exceeding 5% and 8%, respectively. The results were normalized to the protein concentration of each sample.

### Intestinal histomorphology

Jejunal tissue samples fixed in 4% paraformaldehyde were rinsed, dehydrated, and embedded in paraffin. The paraffin-embedded tissue blocks were sectioned at a thickness of 5 μm, mounted on glass slides, and stained with hematoxylin and eosin (**H&E**). Intestinal morphological structures were observed using the RIDET ZERO imaging system (Lei Te Technology Co., Ltd., Shanghai, China). For each section, five intact and well-oriented villi and their associated crypts were randomly selected for measurement. Villus height (**VH**) and crypt depth (**CD**) were determined, and the villus height-to-crypt depth ratio (**VCR**) was subsequently calculated. The mean value of these five measurements per sample was used for statistical analysis.

### Evaluation of transgenerational effects

To evaluate transgenerational effects, fertile eggs from CON and EO breeder groups were collected and incubated for 21 days under standard conditions. Upon hatching, 72 healthy one-day-old chicks per maternal treatment group were randomly selected and allocated to 6 replicate cages (12 chicks per cage) for a 7-day rearing period. Each cage (70 cm × 70 cm × 40 cm) served as a replicate. All offspring were fed a common basal diet (without EO supplementation) with ad libitum access to feed and water to ensure identical rearing environments. Blood samples were obtained via cardiac puncture on day 1 and via the wing vein sinus on day 7. Subsequently, chicks were euthanized by cervical dislocation for the collection of heart, liver, and jejunal tissues. The heart was selected as a representative organ due to its high metabolic demand and susceptibility to oxidative stress during the transition from embryo to hatchling. These samples were processed to analyze immune and antioxidant parameters using the same indices and assay kits as described for the breeder hens.

### Statistical analysis of data

All data were analyzed using SPSS software (IBM SPSS Statistics 27.0, Armonk, NY, USA). Results are expressed as means and SEM. The normality of distribution for each parameter was assessed using the Shapiro-Wilk test, and the homogeneity of variances between the two groups was evaluated using Levene’s test. Accordingly, comparisons between the two treatment groups were performed using the independent samples t-test when both assumptions were met; otherwise, the non-parametric Mann-Whitney U test (Wilcoxon Rank-Sum test) was employed. Statistical significance was declared at *P* < 0.05, while 0.05 ≤ *P* < 0.10 was considered a significant trend. The experimental data structure for parametric analysis is described by the following linear model:

Y_ij_ = μ + T_i_ + ε_ij_

where *Y_ij_* is the observed value for the *j*-th replicate (house) with the *i* th treatment; *μ* is the overall mean; *T_i_* is the fixed effect of the *i* th treatment group (CON or EO); and *ε_ij_* is the random residual error.

## Results

### Reproductive performance

The effects of dietary supplementation with the thymol and eugenol essential oil mixture on the reproductive performance of breeder hens are summarized in Table 1. Dietary EO supplementation significantly increased the percentage of settable eggs (*P* = 0.017). However, no significant differences were observed between the CON and EO groups regarding egg production rate, average egg weight, FCRe, HF, or HE (*P* > 0.05).

**Table 1:** Effects of dietary thymol and eugenol on the reproductive performance of broiler breeders. 1.

Item	Treatments		SEM	*P*-value
	CON	EO		
Laying rate, %	51.8	52.9	1.70	0.645
Percentage of settable eggs, %	96.0 ^b^	96.5 ^a^	0.11	0.017
Average egg weight, g	67.9	67.9	0.20	0.885
FCRe, g:g	2.20	2.21	0.01	0.922
Fertility, %	82.1	82.7	0.80	0.593
HS, %	76.4	77.2	0.92	0.543
HF, %	93.0	93.3	0.24	0.402
HCP, %	87.4	87.8	0.43	0.535

Abbreviations: CON = control, chickens fed a basal diet; EO = essential oil, chickens fed a basal diet containing 12 mg/kg thymol and 4.5 mg/kg eugenol; FCRe = feed conversion ratio for eggs; HS = hatchability of settable eggs; HF = hatchability of fertile eggs; HCP = percentage of healthy day-old chicks.

1 Means are based on 4 replicates per treatment, with 7,500 broiler breeders and 100 eggs per replicate. ^a, b^ Values within a row with different superscripts differ significantly at *P* < 0.05.

### Egg quality

Egg quality data are summarized in Table 2. Dietary supplementation with the EO blend significantly increased eggshell strength (*P* = 0.016), albumen height (*P* = 0.021), and HU (*P* = 0.029). Furthermore, EO treatment decreased eggshell L* (*P* = 0.001) while increasing b* (*P* = 0.046). Other egg quality parameters did not differ significantly (*P* > 0.05).

**Table 2:** Effects of dietary thymol and eugenol on egg quality of broiler breeders. 1.

Item	Treatments		SEM	*P*-value
	CON	EO		
Egg weight, g	63.2	62.3	0.61	0.231
Egg shape index	1.30	1.30	0.01	0.926
Eggshell strength, N	31.1 ^b^	35.4 ^a^	1.18	0.016
Eggshell thickness, mm	0.33	0.33	0.01	0.744
Eggshell color, L*	83.5 ^a^	81.4 ^b^	0.39	0.001
Eggshell color, a*	5.09	5.82	0.29	0.097
Eggshell color, b*	16.6 ^b^	17.9 ^a^	0.45	0.046
Albumen height, mm	5.75 ^b^	6.42 ^a^	0.19	0.021
Yolk color	9.00	9.13	0.15	0.532
Haugh unit	73.1 ^b^	78.7 ^a^	1.71	0.029

Abbreviations: CON = control, chickens fed a basal diet; EO = essential oil, chickens fed a basal diet containing 12 mg/kg thymol and 4.5 mg/kg eugenol; L* = lightness; a* = red-green chromaticity; b* = yellow-blue chromaticity.

1 Means are based on 4 replicates per treatment, with 10 eggs per replicate. ^a, b^ Values within a row with different superscripts differ significantly at *P* < 0.05.

### Serum biochemical indices of breeder chickens

Table 3, Table 4 present the effects of dietary EO supplementation on serum biochemical indices related to inflammation and antioxidant status in breeder hens and roosters. Results indicated that EO significantly increased serum SOD activity in hens (*P* = 0.029). In roosters, EO treatment also significantly elevated serum T-AOC (*P* = 0.006), as well as CAT (*P* = 0.029), SOD (*P* = 0.001), and GSH-Px (*P* = 0.023) activities. However, no significant differences were observed in serum pro-inflammatory cytokine levels between the treatment groups (*P* > 0.05).

**Table 3:** Effects of dietary thymol and eugenol on serum inflammatory and antioxidant parameters of breeding hens. 1.

Item	Treatments		SEM	*P*-value
	CON	EO		
IL-1β, pg/mL	205	172	12.04	0.081
IL-6, pg/mL	24.8	23.8	0.91	0.489
TNF-α, pg/mL	61.3	58.2	2.66	0.433
T-AOC, mmol/L	1.45	1.60	0.07	0.143
GSH, μmol/L	31.9	32.5	1.21	0.733
CAT, U/mL	48.4	51.1	1.15	0.127
SOD, U/mL	149 ^b^	155 ^a^	1.72	0.029
GSH-Px, U/mL	294	332	13.27	0.070

CON = control, chickens fed a basal diet; EO = essential oil, chickens fed a basal diet containing 12 mg/kg thymol and 4.5 mg/kg eugenol; IL-1β = interleukin-1β; IL-6 = interleukin-6; TNF-α = tumor necrosis factor-alpha; T-AOC = total antioxidant capacity; GSH = glutathione; CAT = catalase; SOD = superoxide dismutase; GSH-Px = glutathione peroxidase.

1 Means are based on 4 replicates per treatment, with 3 breeding hens per replicate. ^a, b^ Values within a row with different superscripts differ significantly at *P* < 0.05.

**Table 4:** Effects of dietary thymol and eugenol on serum inflammatory and antioxidant parameters of breeding roosters. 1.

Item	Treatments		SEM	*P*-value
	CON	EO		
IL-1β, pg/mL	247	223	12.14	0.177
IL-6, pg/mL	31.5	31.1	0.70	0.747
TNF-α, pg/mL	69.2	67.7	7.05	0.882
T-AOC, mmol/L	1.30 ^b^	1.89 ^a^	0.12	0.006
GSH, μmol/L	32.9	32.8	1.01	0.964
CAT, U/mL	46.7 ^b^	57.5 ^a^	2.24	0.007
SOD, U/mL	129 ^b^	150 ^a^	1.80	0.001
GSH-Px, U/mL	281 ^b^	317 ^a^	9.50	0.023

CON = control, chickens fed a basal diet; EO = essential oil, chickens fed a basal diet containing 12 mg/kg thymol and 4.5 mg/kg eugenol; IL-1β = interleukin-1β; IL-6 = interleukin-6; TNF-α = tumor necrosis factor-alpha; T-AOC = total antioxidant capacity; GSH = glutathione; CAT = catalase; SOD = superoxide dismutase; GSH-Px = glutathione peroxidase.

1 Means are based on 4 replicates per treatment, with 3 breeding roosters per replicate. ^a, b^ Values within a row with different superscripts differ significantly at *P* < 0.05.

### Biochemical parameters of ovarian and uterine tissue

As shown in Table 5, compared with the CON group, dietary EO supplementation significantly reduced IL-1β levels in ovarian tissue (*P* = 0.005), while other inflammatory and antioxidant parameters remained unaffected (*P* > 0.05).

**Table 5:** Effects of dietary thymol and eugenol on ovarian tissue inflammatory and antioxidant parameters. 1.

Item	Treatments		SEM	*P*-value
	CON	EO		
IL-1β, pg/mg prot	269 ^a^	212 ^b^	11.10	0.005
IL-6, pg/mg prot	28.2	27.8	0.68	0.640
TNF-α, pg/mg prot	58.0	57.4	2.92	0.894
T-AOC, mmol/g prot	30.0	30.6	1.32	0.771
GSH, μmol/g prot	42.5	46.3	2.82	0.373
CAT, U/mg prot	60.0	62.4	2.74	0.537
SOD, U/mg prot	159	160	1.47	0.699
GSH-Px, U/mg prot	222	247	20.00	0.393

CON = control, chickens fed a basal diet; EO = essential oil, chickens fed a basal diet containing 12 mg/kg thymol and 4.5 mg/kg eugenol; IL-1β = interleukin-1β; IL-6 = interleukin-6; TNF-α = tumor necrosis factor-alpha; T-AOC = total antioxidant capacity; GSH = glutathione; CAT = catalase; SOD = superoxide dismutase; GSH-Px = glutathione peroxidase; prot = protein.

1 Means are based on 4 replicates per treatment, with 3 breeding hens per replicate. ^a, b^ Values within a row with different superscripts differ significantly at *P* < 0.05.

In uterine tissue (Table 6), the levels of pro-inflammatory cytokines, including IL-6 (*P* = 0.001), TNF-α (*P* = 0.025), and IL-1β (*P* < 0.001), were significantly lower in the EO group than in the CON group. Concurrently, EO supplementation significantly increased uterine GSH levels (*P* = 0.001) and CAT activity (*P* = 0.003).

**Table 6:** Effects of dietary thymol and eugenol on uterine tissue inflammatory and antioxidant parameters. 1.

Item	Treatments		SEM	*P*-value
	CON	EO		
IL-1β, pg/mg prot	246 ^a^	194 ^b^	4.63	<0.001
IL-6, pg/mg prot	30.4 ^a^	24.6 ^b^	0.88	0.001
TNF-α, pg/mg prot	73.4 ^a^	67.6 ^b^	1.55	0.025
T-AOC, mmol/g prot	70.0	76.3	2.33	0.086
GSH, μmol/g prot	19.5 ^b^	24.3 ^a^	0.69	0.001
CAT, U/mg prot	57.8 ^b^	72.6 ^a^	2.65	0.003
SOD, U/mg prot	150	155	1.66	0.051
GSH-Px, U/mg prot	386	401	15.48	0.499

CON = control, chickens fed a basal diet; EO = essential oil, chickens fed a basal diet containing 12 mg/kg thymol and 4.5 mg/kg eugenol; IL-1β = interleukin-1β; IL-6 = interleukin-6; TNF-α = tumor necrosis factor-alpha; T-AOC = total antioxidant capacity; GSH = glutathione; CAT = catalase; SOD = superoxide dismutase; GSH-Px = glutathione peroxidase; prot = protein.

1 Means are based on 4 replicates per treatment, with 3 breeding hens per replicate. ^a, b^ Values within a row with different superscripts differ significantly at *P* < 0.05.

### Biochemical parameters of jejunal tissue in roosters

The biochemical profiles of jejunal tissue in breeder roosters are presented in Table 7. Compared with the CON group, the EO group exhibited significantly lower jejunal levels of IL-6 (*P* = 0.022) and IL-1β (*P* = 0.033), accompanied by significant increases in T-AOC (*P* = 0.015) and CAT activity (*P* = 0.003).

**Table 7:** Effects of dietary thymol and eugenol on jejunal tissue inflammatory and antioxidant parameters. 1.

Item	Treatments		SEM	*P*-value
	CON	EO		
IL-1β, pg/mg prot	238 ^a^	191 ^b^	13.70	0.033
IL-6, pg/mg prot	32.0 ^a^	29.4 ^b^	0.68	0.022
TNF-α, pg/mg prot	59.4	54.2	2.72	0.206
T-AOC, mmol/g prot	39.8 ^b^	47.3 ^a^	1.83	0.015
GSH, μmol/g prot	37.1	42.6	1.92	0.071
CAT, U/mg prot	49.6 ^b^	60.6 ^a^	2.00	0.003
SOD, U/mg prot	145	143	1.64	0.403
GSH-Px, U/mg prot	388	405	19.45	0.551

CON = control, chickens fed a basal diet; EO = essential oil, chickens fed a basal diet containing 12 mg/kg thymol and 4.5 mg/kg eugenol; IL-1β = interleukin-1β; IL-6 = interleukin-6; TNF-α = tumor necrosis factor-alpha; T-AOC = total antioxidant capacity; GSH = glutathione; CAT = catalase; SOD = superoxide dismutase; GSH-Px = glutathione peroxidase; prot = protein.

1 Means are based on 4 replicates per treatment, with 3 breeding roosters per replicate. ^a, b^ Values within a row with different superscripts differ significantly at *P* < 0.05.

### Biochemical parameters of liver tissue

Table 8 presents the effects of dietary EO supplementation on liver inflammatory and antioxidant parameters in breeder hens and roosters. In hens, dietary EO significantly reduced liver levels of IL-6 (*P* = 0.023) and IL-1β (*P* = 0.020). Conversely, liver T-AOC (*P* = 0.002) and GSH levels (*P* = 0.002), as well as the activities of CAT (*P* = 0.018), SOD (*P* = 0.038), and GSH-Px (*P* = 0.001), were significantly increased in the EO group. Similar trends were observed in roosters, where EO supplementation significantly decreased liver IL-1β levels (*P* = 0.008) and elevated GSH levels (*P* = 0.002) and SOD (*P* = 0.011) and GSH-Px (*P* = 0.009) activities.

**Table 8:** Effects of dietary thymol and eugenol on liver tissue inflammatory and antioxidant parameters. 1.

Item	Treatments		SEM	*P*-value
	CON	EO		
Hen				
IL-1β, pg/mg prot	246 ^a^	200 ^b^	11.87	0.020
IL-6, pg/mg prot	28.5 ^a^	25.1 ^b^	0.91	0.023
TNF-α, pg/mg prot	248	244	21.73	0.903
T-AOC, mmol/g prot	37.9 ^b^	47.9 ^a^	1.70	0.002
GSH, μmol/g prot	32.0 ^b^	46.1 ^a^	2.32	0.002
CAT, U/mg prot	51.4 ^b^	56.8 ^a^	1.37	0.018
SOD, U/mg prot	147 ^b^	155 ^a^	2.28	0.038
GSH-Px, U/mg prot	403 ^b^	535 ^a^	20.56	0.001
Rooster				
IL-1β, pg/mg prot	269 ^a^	209 ^b^	12.83	0.008
IL-6, pg/mg prot	29.8	29.9	1.90	0.984
TNF-α, pg/mg prot	62.2	60.3	4.88	0.785
T-AOC, mmol/g prot	29.0	30.8	0.83	0.147
GSH, μmol/g prot	30.9 ^b^	36.4 ^a^	0.95	0.002
CAT, U/mg prot	55.6	60.8	2.99	0.245
SOD, U/mg prot	148 ^a^	140 ^b^	1.81	0.011
GSH-Px, U/mg prot	383 ^b^	439 ^a^	12.34	0.009

CON = control, chickens fed a basal diet; EO = essential oil, chickens fed a basal diet containing 12 mg/kg thymol and 4.5 mg/kg eugenol; IL-1β = interleukin-1β; IL-6 = interleukin-6; TNF-α = tumor necrosis factor-alpha; T-AOC = total antioxidant capacity; GSH = glutathione; CAT = catalase; SOD = superoxide dismutase; GSH-Px = glutathione peroxidase; prot = protein.

1 Means are based on 4 replicates per treatment, with 3 breeding hens or breeding roosters per replicate. ^a, b^ Values within a row with different superscripts differ significantly at *P* < 0.05.

### Jejunal morphology

The effects of dietary EO on the jejunal morphology of breeder hens are summarized in Table 9. Compared with the CON group, hens in the EO group exhibited significantly increased villus height (*P* = 0.002) and VCR (*P* = 0.012). No significant differences were observed in crypt depth between the treatment groups (*P* > 0.05).

**Table 9:** Effects of dietary thymol and eugenol on jejunum tissue histomorphology of broiler breeders. 1.

Item	Treatments		SEM	*P*-value
	CON	EO		
VH, μm	1158 ^b^	1518 ^a^	61.80	0.002
CD, μm	244	223	18.65	0.453
VCR	4.90 ^b^	6.96 ^a^	0.47	0.012

CON = control, chickens fed a basal diet; EO = essential oil, chickens fed a basal diet containing 12 mg/kg thymol and 4.5 mg/kg eugenol; VH = villus height; CD = crypt depth; VCR = villus height-to-crypt depth ratio.

1 Means are based on 4 replicates per treatment, with 3 breeding hens per replicate. ^a, b^ Values within a row with different superscripts differ significantly at *P* < 0.05.

### Effects of maternal EO supplementation on offspring chicks

***Production performance.*** The production performance of 7-day-old offspring chicks is summarized in Table 10. Compared with the CON group, the ADFI of chicks in the EO group was significantly reduced (*P* = 0.036). However, no significant differences were observed in hatch weight, 7-d BW, ADG, or FCR between the two groups (*P* > 0.05).

**Table 10:** Effects of dietary thymol and eugenol on production performance of day-old chicks at 7 days post-hatch. 1.

Item	Treatments		SEM	*P*-value
	CON	EO		
Hatch weight, g	45.5	45.7	0.48	0.390
7-d BW, g	160	155	7.22	0.162
ADFI, g	30.4 ^a^	27.4 ^b^	0.90	0.036
ADG, g	16.5	15.6	0.39	0.141
FCR, g:g	1.60	1.49	0.06	0.248

CON = control, chickens fed a basal diet; EO = essential oil, chickens fed a basal diet containing 12 mg/kg thymol and 4.5 mg/kg eugenol; BW = body weight; ADFI = average daily feed intake; ADG = average daily gain; FCR = feed conversion ratio.

1 Means are based on 6 replicates per treatment, with 12 chicks per replicate. ^a, b^ Values within a row with different superscripts differ significantly at *P* < 0.05.

***Serum biochemical indices of chicks.*** Serum biochemical parameters of chicks at 1 and 7 days of age are shown in Table 11. At 1 day of age, serum levels of the pro-inflammatory cytokines IL-1β (*P* = 0.019) and TNF-α (*P* < 0.001) were significantly lower in the EO group than in the CON group. Conversely, GSH levels (*P* = 0.021) and GSH-Px activity (*P* = 0.002) were significantly elevated. By 7 days of age, serum IL-1β (*P* = 0.006), IL-6 (*P* = 0.013), and TNF-α (*P* = 0.026) remained significantly lower in the EO group, while SOD (*P* = 0.002) and GSH-Px (*P* < 0.001) activities were significantly higher.

**Table 11:** Effects of dietary thymol and eugenol on serum inflammatory and antioxidant parameters of chicks. 1.

Item	Treatments		SEM	*P*-value
	CON	EO		
1-day-old				
IL-1β, pg/mL	294 ^a^	253 ^b^	10.20	0.019
IL-6, pg/mL	29.1	26.3	2.85	0.508
TNF-α, pg/mL	107 ^a^	75.8 ^b^	2.19	< 0.001
T-AOC, mmol/L	2.28	2.42	0.14	0.507
GSH, μmol/L	34.0 ^b^	41.1 ^a^	1.78	0.021
CAT, U/mL	45.9	50.0	2.61	0.315
SOD, U/mL	130	132	2.09	0.504
GSH-Px, U/mL	247 ^b^	301 ^a^	9.37	0.002
7-day-old				
IL-1β, pg/mL	324 ^a^	263 ^b^	12.41	0.006
IL-6, pg/mL	36.7 ^a^	32.1 ^b^	1.07	0.013
TNF-α, pg/mL	67.7 ^a^	60.2 ^b^	2.04	0.026
T-AOC, mmol/L	1.69	1.83	0.15	0.523
GSH, μmol/L	33.8	38.1	2.22	0.205
CAT, U/mL	31.5	39.1	3.40	0.144
SOD, U/mL	117 ^b^	135 ^a^	3.16	0.002
GSH-Px, U/mL	276 ^b^	346 ^a^	6.38	< 0.001

CON = control, chickens fed a basal diet; EO = essential oil, chickens fed a basal diet containing 12 mg/kg thymol and 4.5 mg/kg eugenol; IL-1β = interleukin-1β; IL-6 = interleukin-6; TNF-α = tumor necrosis factor-alpha; T-AOC = total antioxidant capacity; GSH = glutathione; CAT = catalase; SOD = superoxide dismutase; GSH-Px = glutathione peroxidase.

1 Means are based on 6 replicates per treatment, with 2 chicks per replicate. ^a, b^ Values within a row with different superscripts differ significantly at *P* < 0.05.

***Biochemical indices in the liver of chicks.*** Table 12 presents the liver biochemical parameters of offspring chicks. On day 1, liver IL-1β levels in the EO group were significantly decreased (*P* = 0.004), while CAT activity was significantly increased (*P* = 0.001). On day 7, liver IL-1β levels remained significantly lower (*P* = 0.017), accompanied by a concurrent increase in SOD activity (*P* = 0.025).

**Table 12:** Effects of dietary thymol and eugenol on liver tissue inflammatory and antioxidant parameters of chicks. 1.

Item	Treatments		SEM	*P*-value
	CON	EO		
1-day-old				
IL-1β, pg/mg prot	256 ^a^	206 ^b^	9.62	0.004
IL-6, pg/mg prot	27.3	26.1	1.50	0.563
TNF-α, pg/mg prot	65.1	60.0	5.13	0.496
T-AOC, mmol/g prot	36.7	42.9	4.79	0.377
GSH, μmol/g prot	27.8	33.7	2.53	0.127
CAT, U/mg prot	32.4 ^b^	51.9 ^a^	1.18	0.001
SOD, U/mg prot	34.9	36.8	1.02	0.231
GSH-Px, U/mg prot	365	420	20.63	0.087
7-day-old				
IL-1β, pg/mg prot	298 ^a^	241 ^b^	14.10	0.017
IL-6, pg/mg prot	31.4 ^a^	27.2 ^b^	1.59	0.034
TNF-α, pg/mg prot	42.1 ^a^	30.2 ^b^	3.83	0.050
T-AOC, mmol/g prot	28.6	29.1	1.61	0.842
GSH, μmol/g prot	29.3	33.4	4.33	0.517
CAT, U/mg prot	47.2	50.8	1.65	0.155
SOD, U/mg prot	35.9 ^b^	38.1 ^a^	0.60	0.025
GSH-Px, U/mg prot	382	393	21.92	0.303

CON = control, chickens fed a basal diet; EO = essential oil, chickens fed a basal diet containing 12 mg/kg thymol and 4.5 mg/kg eugenol; IL-1β = interleukin-1β; IL-6 = interleukin-6; TNF-α = tumor necrosis factor-alpha; T-AOC = total antioxidant capacity; GSH = glutathione; CAT = catalase; SOD = superoxide dismutase; GSH-Px = glutathione peroxidase; prot = protein.

1 Means are based on 6 replicates per treatment, with 2 chicks per replicate. ^a, b^ Values within a row with different superscripts differ significantly at *P* < 0.05.

***Biochemical indices in the heart of chicks.*** The inflammatory and antioxidant parameters in heart tissue are shown in Table 13. On both day 1 and day 7, the heart IL-1β levels in the EO group were significantly lower than those in the CON group (*P* = 0.014 and *P* = 0.009, respectively). On day 1, heart T-AOC (*P* = 0.001), GSH levels (*P* < 0.001), and GSH-Px activity (*P* = 0.007) were all significantly increased in the EO group. By day 7, no significant differences in heart antioxidant parameters were observed between groups (*P* > 0.05).

**Table 13:** Effects of dietary thymol and eugenol on heart tissue inflammatory and antioxidant parameters of chicks. 1.

Item	Treatments		SEM	*P*-value
	CON	EO		
1-day-old				
IL-1β, pg/mg prot	296 ^a^	255 ^b^	9.53	0.014
IL-6, pg/mg prot	28.3	27.9	2.06	0.889
TNF-α, pg/mg prot	53.5	50.1	1.91	0.273
T-AOC, mmol/g prot	40.1 ^b^	59.2 ^a^	1.42	0.001
GSH, μmol/g prot	66.9 ^b^	79.7 ^a^	1.45	0.001
CAT, U/mg prot	62.2	63.9	3.06	0.704
SOD, U/mg prot	40.4	39.2	1.32	0.547
GSH-Px, U/mg prot	376 ^b^	399 ^a^	4.88	0.007
7-day-old				
IL-1β, pg/mg prot	294 ^a^	233 ^b^	14.19	0.009
IL-6, pg/mg prot	32.1	28.9	1.64	0.195
TNF-α, pg/mg prot	62.2	59.5	5.08	0.708
T-AOC, mmol/g prot	56.4	64.2	2.71	0.066
GSH, μmol/g prot	62.5	67.8	2.81	0.218
CAT, U/mg prot	58.8	61.2	5.42	0.757
SOD, U/mg prot	32.8	34.4	1.37	0.422
GSH-Px, U/mg prot	388	399	16.00	0.639

CON = control, chickens fed a basal diet; EO = essential oil, chickens fed a basal diet containing 12 mg/kg thymol and 4.5 mg/kg eugenol; IL-1β = interleukin-1β; IL-6 = interleukin-6; TNF-α = tumor necrosis factor-alpha; T-AOC = total antioxidant capacity; GSH = glutathione; CAT = catalase; SOD = superoxide dismutase; GSH-Px = glutathione peroxidase; prot = protein.

1 Means are based on 6 replicates per treatment, with 2 chicks per replicate. ^a, b^ Values within a row with different superscripts differ significantly at *P* < 0.05.

***Biochemical indices in the jejunum of chicks.*** Jejunal biochemical parameters of chicks are presented in Table 14. At 1 day of age, the jejunal IL-1β level was significantly decreased (*P* < 0.001), while CAT activity was significantly increased (*P* = 0.035) in the EO group. At 7 days of age, the jejunal IL-1β level remained significantly lower (*P* = 0.011), and GSH-Px activity was significantly higher (*P* = 0.002) in the EO group.

**Table 14:** Effects of dietary thymol and eugenol on jejunal tissue inflammatory and antioxidant parameters of chicks. 1.

Item	Treatments		SEM	*P*-value
	CON	EO		
1-day-old				
IL-1β, pg/mg prot	193 ^a^	136 ^b^	6.43	< 0.001
IL-6, pg/mg prot	30.2	29.7	1.78	0.847
TNF-α, pg/mg prot	51.8	49.0	3.59	0.600
T-AOC, mmol/g prot	29.6	30.4	0.85	0.522
GSH, μmol/g prot	62.7	67.2	2.05	0.159
CAT, U/mg prot	56.4 ^b^	60.5 ^a^	1.18	0.035
SOD, U/mg prot	36.9	38.9	0.90	0.151
GSH-Px, U/mg prot	360	409	17.64	0.074
7-day-old				
IL-1β, pg/mg prot	279 ^a^	239 ^b^	9.03	0.011
IL-6, pg/mg prot	27.3	26.6	0.84	0.566
TNF-α, pg/mg prot	46.7	46.4	3.02	0.950
T-AOC, mmol/g prot	26.3	29.3	1.33	0.139
GSH, μmol/g prot	64.7	66.8	1.90	0.464
CAT, U/mg prot	39.9	42.7	2.13	0.371
SOD, U/mg prot	35.6	38.2	0.97	0.082
GSH-Px, U/mg prot	349 ^b^	391 ^a^	7.25	0.002

CON = control, chickens fed a basal diet; EO = essential oil, chickens fed a basal diet containing 12 mg/kg thymol and 4.5 mg/kg eugenol; IL-1β = interleukin-1β; IL-6 = interleukin-6; TNF-α = tumor necrosis factor-alpha; T-AOC = total antioxidant capacity; GSH = glutathione; CAT = catalase; SOD = superoxide dismutase; GSH-Px = glutathione peroxidase; prot = protein.

1 Means are based on 6 replicates per treatment, with 2 chicks per replicate. ^a, b^ Values within a row with different superscripts differ significantly at *P* < 0.05.

## Discussion

Since the prohibition of antibiotic use in animal feed, the metabolic load and nutritional stress induced by the high egg-laying performance of breeder hens have become major challenges in poultry production (Korver, 2023). Essential oils (EOs) derived from plants are regarded as growth-promoting (Zhang et al., 2014) and antibacterial (Bassolé and Juliani, 2012) additives in poultry production due to their diverse pharmacological activities, such as antioxidant effects, inhibition of microbial growth, and alleviation of metabolic stress (Semeniuc et al., 2017). Thymol and eugenol are two natural plant essential oil components with excellent biological activities, including antioxidant, anti-inflammatory, and immunomodulatory properties. They are widely present in plants such as thyme and clove and have become important alternatives to traditional antibiotics in poultry farming (Latorre et al., 2025). While synergistic effects of combined EOs have been suggested (Jaradat et al., 2017), research on their combined application regarding breeder reproductive performance and transgenerational effects remains limited. Therefore, this study evaluated the impact of thymol and eugenol on the reproductive performance, egg quality, and antioxidant capacity of breeder hens, as well as the health status of their progeny.

In the current study, we observed that EO supplementation significantly improved eggshell strength, albumen height, and Haugh units, aligning with Aljuwayd et al. (2023). The improvement in eggshell strength may be attributed to enhanced antioxidant capacity in the uterus and improved intestinal mineral absorption. The antioxidant properties of thymol and eugenol are known to increase the activity of antioxidant enzymes in uterine tissues, thereby preserving the functional integrity of carbonic anhydrase II (CAII) and calcium-binding proteins in the shell gland, which are critical determinants of eggshell quality (Liu et al., 2020). Furthermore, the anti-inflammatory properties of these essential oils may alleviate physiological stress in the reproductive tract, facilitating a more uniform deposition of the calcium carbonate matrix (Basiouni et al., 2023). This, in turn, contributes to the maintenance of a stable eggshell microstructure and high mechanical strength. Notably, the increase in the percentage of settable eggs without a change in the total laying rate suggests a reduction in deformed or broken eggs, which may be a direct consequence of this optimized uterine microenvironment (Zhang et al., 2019; Jing et al., 2022). Similarly, the beneficial effects on albumen height and Haugh units may stem from enhanced nutrient absorption and antioxidant defense (Mnisi et al., 2023), as oxidative stress is established to impair both eggshell formation and albumen quality (Sahin et al., 2003).

Regarding egg production, our results indicated that dietary thymol and eugenol did not exert significant effects on laying rate, average egg weight, or FCRe. This is in agreement with the findings of Wang et al. (2022), who reported that dietary essential oil supplementation had no adverse impact on the laying rate of breeder hens. In terms of reproductive performance, we observed that the EO blend did not significantly alter fertility or hatchability, which is consistent with the findings of Luna et al. (2012). Furthermore, the lack of adverse effects on fertility and hatchability indicates that this EO blend maintains a high safety profile regarding reproductive traits (Adewoyin et al., 2017; Alagawany et al., 2018), unlike certain fatty acids that may directly alter fecundity (Surai et al., 2017).

In the present study, we found that dietary supplementation with thymol and eugenol significantly reduced IL-1β levels in the ovarian tissue and decreased the levels of IL-1β, IL-6, and TNF-α in the uterine tissue of breeder hens. These cytokines are recognized as key pro-inflammatory mediators in the systemic and local inflammatory response (Liu et al., 2019). The reduction of these factors is of great physiological significance, as IL-1β is a key cytokine associated with follicular atresia (Field et al., 2014), and its sustained high expression in the uterus can induce inflammation, thereby impairing eggshell formation and oviduct health (Patel et al., 2017; Velez et al., 2021). Therefore, by suppressing IL-1β expression, this EO blend helps improve the ovarian and uterine microenvironment, supporting optimal follicular development and egg quality (Basiouni et al., 2023).

Furthermore, our results showed that IL-1β levels were also significantly reduced in the liver tissues of both breeder roosters and hens, suggesting that these two essential oils possess the potential to modulate systemic inflammatory signaling pathways (Liñán-Atero et al., 2024; Latorre et al., 2025). These findings align with multiple studies supporting the immunomodulatory activities of thymol and eugenol. For instance, Lanzarin et al. (2023) reported that thymol alleviated the inflammatory response and enhanced antioxidant capacity in juvenile zebrafish. Similarly, eugenol has been shown to exert anti-inflammatory effects in mouse models by activating the PPAR-γ pathway (Huang et al., 2025). Collectively, the results of this study indicate that supplementation with these two essential oils normalized the levels of pro-inflammatory factors across multiple tissues, suggesting their pivotal role in alleviating the chronic inflammatory state typically associated with high-intensity egg production.

A key outcome of the dietary EO intervention was the robust enhancement of cellular antioxidant defense, characterized by significantly increased SOD, CAT, and GSH-Px activities, alongside elevated T-AOC and GSH levels in breeder tissues. These parameters serve as well-established biomarkers for assessing cellular antioxidant capacity and redox homeostasis (Dharshini et al., 2023). Physiologically, this reinforcement of the antioxidant system is intimately coupled with the anti-inflammatory properties of thymol and eugenol. High-density rearing and intense egg production typically subject breeder hens to substantial metabolic loads, during which inflammation and oxidative stress operate as interconnected, mutually reinforcing processes (Biswas, 2016; Widowski et al., 2022). Specifically, pro-inflammatory cytokines, including IL-1β and TNF-α, can upregulate NADPH oxidase expression, thereby driving the generation of reactive oxygen species (ROS) (Liu et al., 2025). Accumulating ROS in tissues further accelerates oxidative damage, fostering a destructive positive feedback loop with the local inflammatory response (Ding et al., 2023). At the molecular level, the crosstalk between the classical inflammatory pathway (NF-κB) and the antioxidant pathway (Nrf2) governs this vicious cycle (Baiskhanova and Schäfer, 2024). Overactivation of NF-κB not only promotes the transcription of pro-inflammatory mediators but also actively suppresses Nrf2 signaling, thereby undermining downstream cellular antioxidant defenses (Banaszak et al., 2024). Given this antagonistic relationship, the antioxidant improvements observed here are highly likely secondary to the potent primary anti-inflammatory activities of thymol and eugenol. These findings closely align with previous reports by Magalhães et al. (2019) and Gulec (2021), which demonstrated that eugenol can synergistically alleviate oxidative stress and inflammation by reinforcing SOD activity and suppressing TNF-α and IL-1β levels.

Dietary supplementation with thymol and eugenol significantly enhanced the intestinal morphology of breeder chickens, as evidenced by the increased villus height and VCR. These two parameters serve as key morphological indicators for assessing gastrointestinal development and mucosal architecture; higher villi and an increased VCR substantially expand the intestinal absorptive surface area, thereby reflecting superior feed utilization efficiency and nutrient absorption capacity (Kang et al., 2017; Zhang et al., 2023). Structurally and functionally, a healthy intestine is paramount to ensure efficient nutrient assimilation capable of meeting the high metabolic and energy demands of breeder hens, which is crucial for maintaining a stable laying rate, hatchability, and egg quality (Zhang et al., 2022). Given that gut morphology is not only a direct factor influencing nutrient uptake but is also intricately associated with systemic immune responses and overall performance (Jha et al., 2019; Zhang et al., 2022; Liu and Kim, 2023), the optimization of the intestinal tract observed here provides a robust physiological foundation for the birds. These morphological improvements closely align with prior research indicating that plant-derived essential oils can ameliorate intestinal structure and indirectly support the reproductive performance of poultry (Kwon et al., 2019; Damasceno et al., 2024). Mechanistically, this gut-stabilizing effect may also be tied to enhanced barrier integrity and attenuated localized inflammation. For instance, Omonijo et al. (2019) demonstrated that thymol upregulates the expression of tight junction proteins, such as zonula occludens-1 (ZO-1) and actin, in intestinal epithelial cells, thereby reinforcing intestinal barrier function. Collectively, our results suggest that alongside their direct antioxidant and anti-inflammatory bioactivities in reproductive tissues, thymol and eugenol exert an additional layer of reproductive support by mitigating metabolic stress through the prospective amelioration of gut health.

Maternal dietary supplementation with the thymol and eugenol blend exerted a pronounced transgenerational benefit, significantly suppressing early-life pro-inflammatory cytokines (IL-1β, IL-6, and TNF-α) while synchronously reinforcing CAT, SOD, and GSH-Px enzyme activities in the offspring chicks. These outcomes demonstrate that the combined essential oils successfully upgrade the systemic anti-inflammatory capacity and metabolic resilience of the progeny by enhancing the corresponding maternal health and antioxidant status. Structurally, the realization of such a transgenerational strategy relies heavily on the maternal transfer of genetic material, antibodies, and critical protective nutrients into the egg during formation (Xu et al., 2025). Because the initial antioxidant defense and free radical scavenging efficiency of neonates are inextricably linked to the physiological status of the breeder hens (Alagawany et al., 2020), optimizing the maternal microenvironment provides a vital buffer for the chicks during their highly vulnerable post-hatch period. Our findings firmly support the established poultry husbandry concept that maternal nutritional intervention serves as an effective programming approach to optimize early-life resilience in the subsequent generation (Surai and Kochish, 2019b). Similar carry-over effects have been well-documented in parental diets enriched with vitamin E, N-acetylcysteine, or plant polyphenols, which concurrently improve the feed conversion ratio, anti-inflammatory capacity, and antioxidant status across generations (Lv et al., 2018; İpçak and Denli, 2024). Ultimately, the synchronized improvement in both maternal and progeny health parameters observed here confirms that this essential oil combination serves as an effective maternal programming agent, aligning with the transgenerational paradigms established in previous literature (Surai et al., 2019a).

**Fig. 1:**
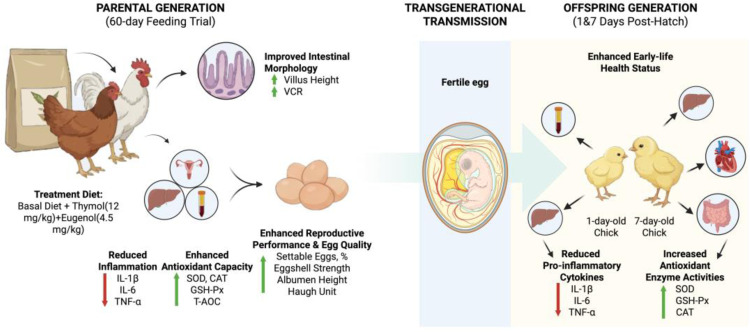
Graphical representation of the experimental design and the impact of maternal thymol and eugenol supplementation. The diagram illustrates the dietary intervention in broiler breeders and the subsequent improvements in reproductive performance, egg quality, and the systemic antioxidant and anti-inflammatory status of both parents and their offspring at 1 and 7 days post-hatch. (↑) indicates a significant increase, and (↓) indicates a significant decrease compared to the CON group (*P* < 0.05).

In summary, our findings outline a comprehensive conceptual model illustrating the transgenerational benefits derived from maternal thymol and eugenol supplementation (Fig. 1). Dietary intake of these essential oil compounds optimizes the nutritional and immunological microenvironment within the fertile egg by mitigating systemic and reproductive tract inflammation, while simultaneously fortifying the antioxidant defense system in the breeders. This maternal nutritional programming, in turn, confers a sustained 'carry-over effect' on the progeny, characterized by attenuated pro-inflammatory responses and enhanced redox homeostasis during the critical first week post-hatch. Consequently, the offspring exhibit heightened resilience against environmental stressors and potential pathological challenges. Collectively, this strategic nutritional intervention successfully links parental health with early-life resilience in the progeny, constituting a crucial and sustainable approach for modern poultry health management (Alkie et al., 2019).

## Conclusion

In conclusion, dietary supplementation with a mixture of thymol and eugenol significantly improved the percentage of settable eggs and egg quality of breeder hens by enhancing intestinal morphology, modulating immune function, and alleviating oxidative stress in the liver, heart, and reproductive tissues. With the laying rate and fertility remaining stable, the increase in the number of settable eggs effectively enhanced the overall reproductive efficiency of the breeders. Furthermore, maternal EO supplementation effectively fortified the antioxidant defense system and suppressed pro-inflammatory responses in offspring chicks during the critical post-hatch period. These findings demonstrate that the thymol-eugenol combination exerts potent transgenerational effects through maternal nutritional programming, offering a sustainable strategy to optimize the health and resilience of both breeder hens and their progeny.

## **Disclosures**

We declare that we have no financial and personal relationships with other people or organizations that can inappropriately influence our work, and there is no professional or other personal interest of any nature or kind in any product, service and/or company that could be construed as influencing the content of this paper.

## CRediT authorship contribution statement

**Haojian Sun:** Writing – original draft, Investigation. **Peng Sun:** Investigation, Data curation. **Xinran Zhang:** Data curation. **Okasha Hamada:** Writing – review & editing. **Linglian Kong:** Writing – review & editing. **Zhigang Song:** Supervision, Funding acquisition, Conceptualization.

## Appendix Supplementary materials

Image, application 1

## Footnotes

Supplementary material associated with this article can be found, in the online version, at doi:10.1016/j.psj.2026.107193.
