# Supplementary material for: Effects of thymol and eugenol supplementation on reproductive performance, egg quality, and offspring health in broiler breeders
Source: Poult Sci. 2026 May 27;105(9):107193. doi: 10.1016/j.psj.2026.107193 (PMC13273655; doi:10.1016/j.psj.2026.107193)
Supplement: Supplementary file 1 [file mmc1.docx]

**Effects of thymol and eugenol supplementation on reproductive performance, egg quality, and offspring health in broiler breeders**

Haojian Sun ^a^, Peng Sun ^a^, Xinran Zhang ^a^, Okasha Hamada ^a, b^, Linglian Kong ^c^, Zhigang Song ^a,^ *

**Supplementary Table S1**

Ingredient composition and nutritional components of the basal diet (%, air- dry basis)

| Item | Content |
| --- | --- |
| Ingredients |  |
| Corn | 54.89 |
| Soybean meal, 46% | 13.60 |
| Wheat flour | 10.00 |
| Wheat germ flour, 25% | 8.00 |
| Salt | 0.35 |
| Limestone, 37% | 8.50 |
| Dicalcium phosphate | 1.45 |
| Soybean oil | 2.40 |
| Vitamin premix^1^ | 0.25 |
| Mineral premix^2^ | 0.10 |
| Choline chloride, 50% | 0.15 |
| Methionine, 99% | 0.17 |
| Lysine, 70% | 0.02 |
| Threonine, 98.5% | 0.07 |
| Tyrosine, 30% | 0.05 |
| Total | 100.00 |
| Nutritional composition^3^ |  |
| Metabolizable energy, kJ/kg | 11924.4 |
| Crude protein | 14.50 |
| Lysine | 0.60 |
| Methionine | 0.38 |
| Calcium | 3.60 |
| Available phosphorus | 0.33 |

^1^ Provided for per kilogram of diet: vitamin A, 12,000 IU/kg; vitamin D3, 5,000 IU/kg; vitamin E, 88 IU/kg; vitamin K3, 3.2 mg; vitamin B1, 3.2 mg; vitamin B2, 8.6 mg; nicotinic acid, 65 mg; pantothenic acid, 20 mg; vitamin B6, 4.3 mg; biotin, 0.22 mg; folic acid, 2.2 mg; vitamin B12, 0.017 mg.

^2^ Provided for per kilogram of diet: I, 1.25 mg; Fe, 20 mg; Mn, 120 mg; Se, 0.3 mg; Zn, 110 mg.

^3^ The levels of crude protein, lysine, methionine, calcium and available phosphorus were analyzed, while metabolizable energy was calculated.
